# Supplementary material for: Structure-Based Bioisosterism Design, Synthesis, Biological Evaluation and In Silico Studies of Benzamide Analogs as Potential Anthelmintics
Source: Molecules. 2022 Apr 20;27(9):2659. doi: 10.3390/molecules27092659 (PMC9102753; doi:10.3390/molecules27092659)
Supplement: Supplementary file 1 [file molecules-27-02659-s001.zip › molecules-1660620-supplementary.pdf]

# Supporting Information

## Structure-based bioisosterism design, synthesis, biological evaluation and *in silico* studies of benzamide analogs as potential anthelmintics.

**Franco Vairoletti[1,2], Margot Paulino[3], Graciela Mahler[1], Gustavo Salinas[4,*] and Cecilia Saiz[1,*]**

[1] Laboratorio de Química Farmacéutica, DQO, Facultad de Química, Universidad de la República (UdelaR), Montevideo, Uruguay; fvairoletti@fq.edu.uy.
[2] Graduate Program in Chemistry, Facultad de Química, UdelaR, Montevideo, 11800, Uruguay.
[3] LaBioFarMol, DETEMA, Facultad de Química, UdelaR, Montevideo, Uruguay; margot@fq.edu.uy
[4] Laboratorio de Biología de Gusanos. Unidad Mixta, Departamento de Biociencias, Facultad de Química, Universidad de la República - Institut Pasteur de Montevideo, Montevideo, Uruguay; gsalin@fq.edu.uy.
* Correspondence: gsalin@fq.edu.uy and csaiz@fq.edu.uy; Tel.: (GS +598 2522 0910 and CS +598 29290290).

## Table of contents:

1. Compounds **2, 3, 4, 5a, 6, 7, 8c and 9b-d**: $^{1}H$ and $^{13}C$ NMR spectra.
2. Synthesis and characterization of compounds: **9a**, **10b**, **11a**, **11b** and **11c**.
3. Docking optimization.
4. Docking poses obtained for Wact-11 bioisostere derivatives **2-8c**.
5. Interaction diagrams for compounds **3** and **4.**
6. Fluopyram in its docking pose with the inner mitochondrial membrane modeled.
7. *C. elegans* L1 motility assay results for compounds **8a, 8c, 9a, 9c** and **9d.**

$^{1}$H-NMR ($CDCl_3$ with 0.05% TMS, 400 MHz)

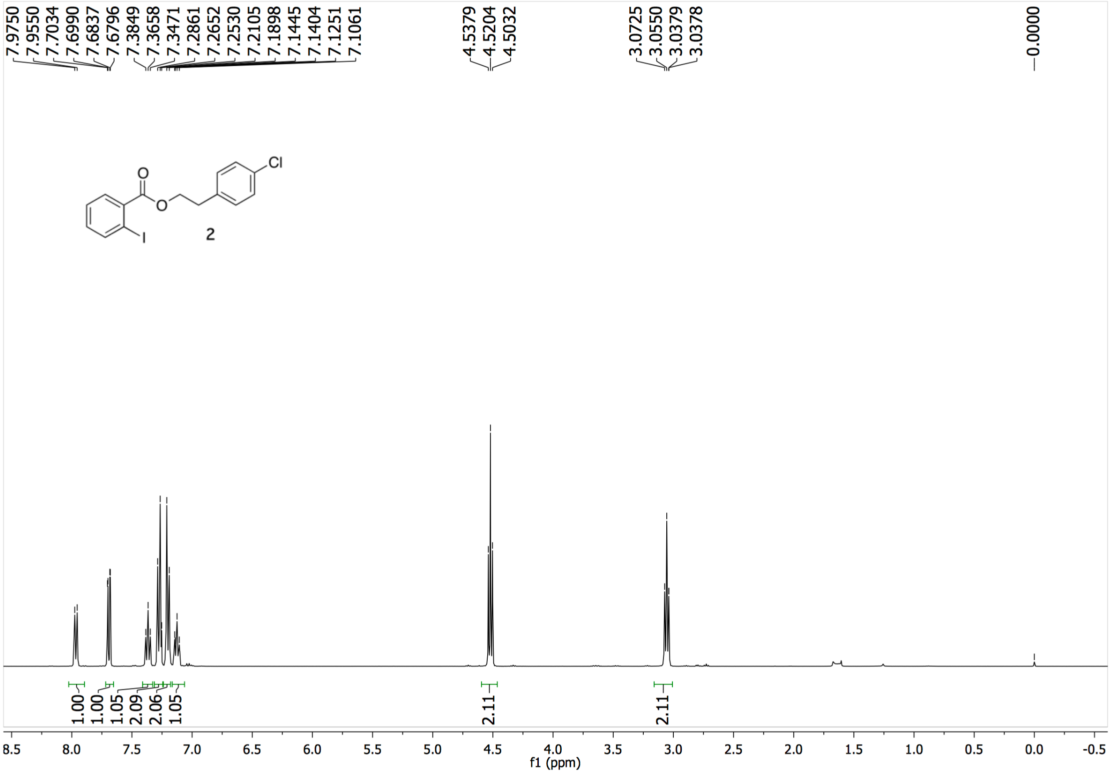


$^{13}$C-NMR ($CDCl_3$ with 0.05% TMS, 100 MHz)

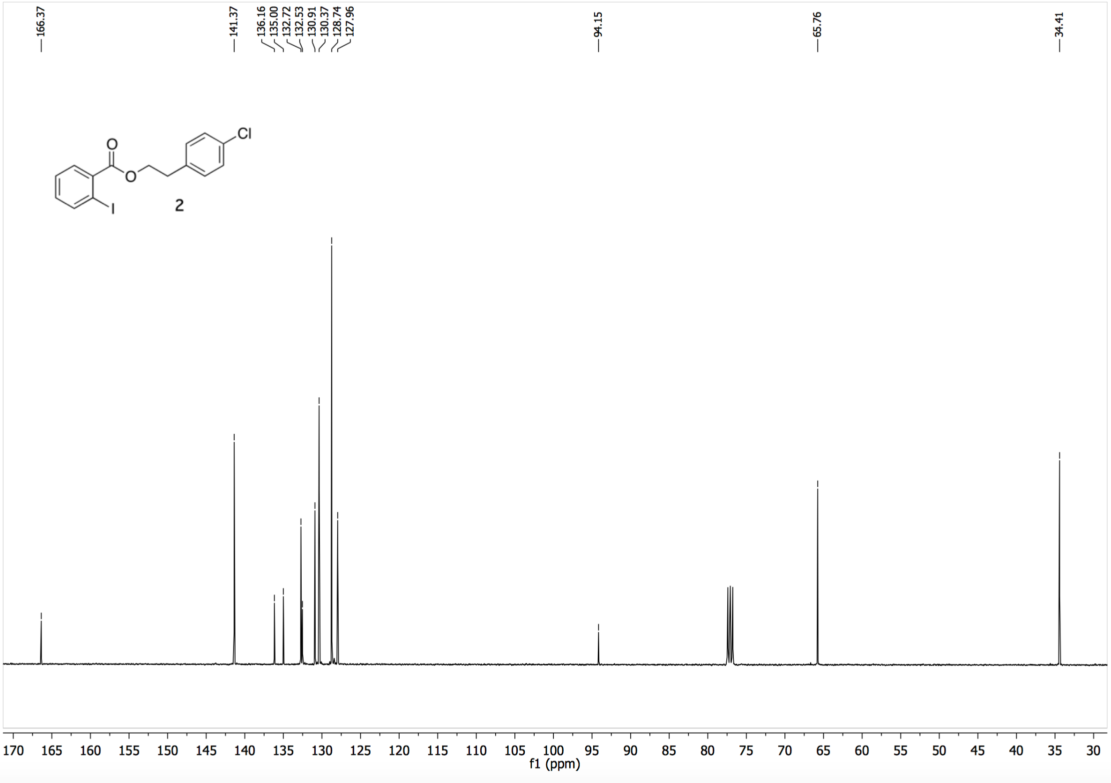

$^1$H-NMR ($CDCl_3$ with 0.05% TMS, 400 MHz)

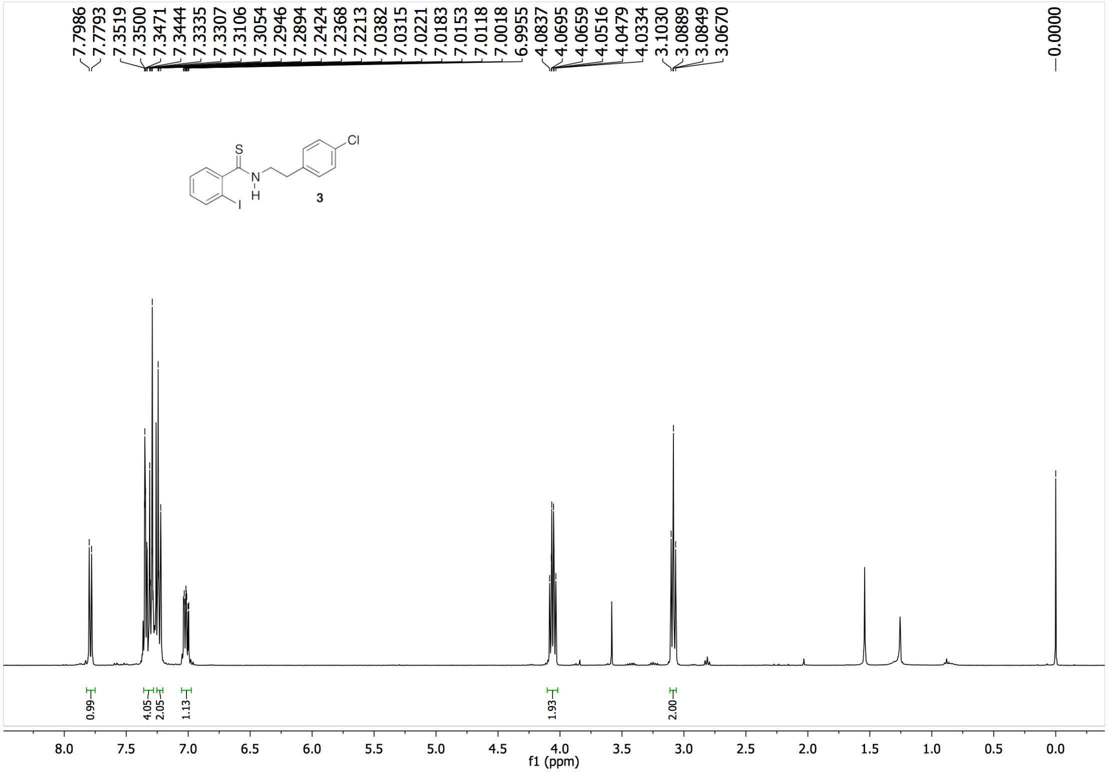


$^{13}$C-NMR ($CDCl_3$ with 0.05% TMS, 100 MHz)

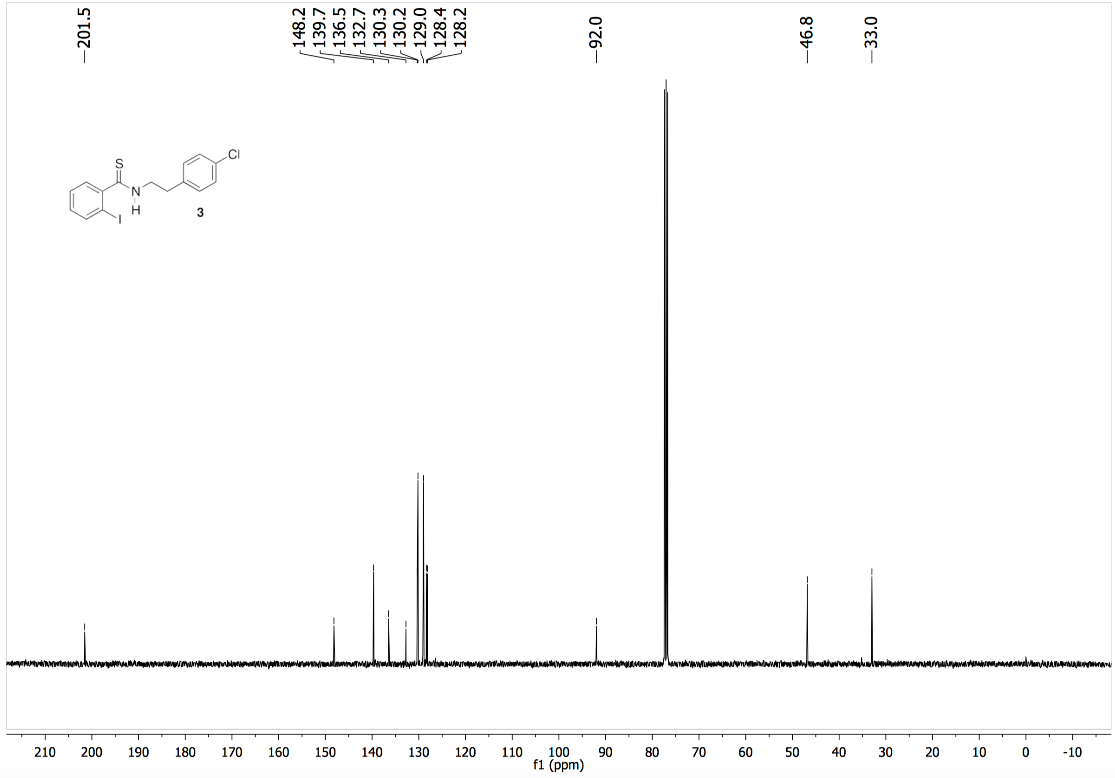

$^{1}$H-NMR ($CDCl_3$ with 0.05% TMS, 400 MHz)

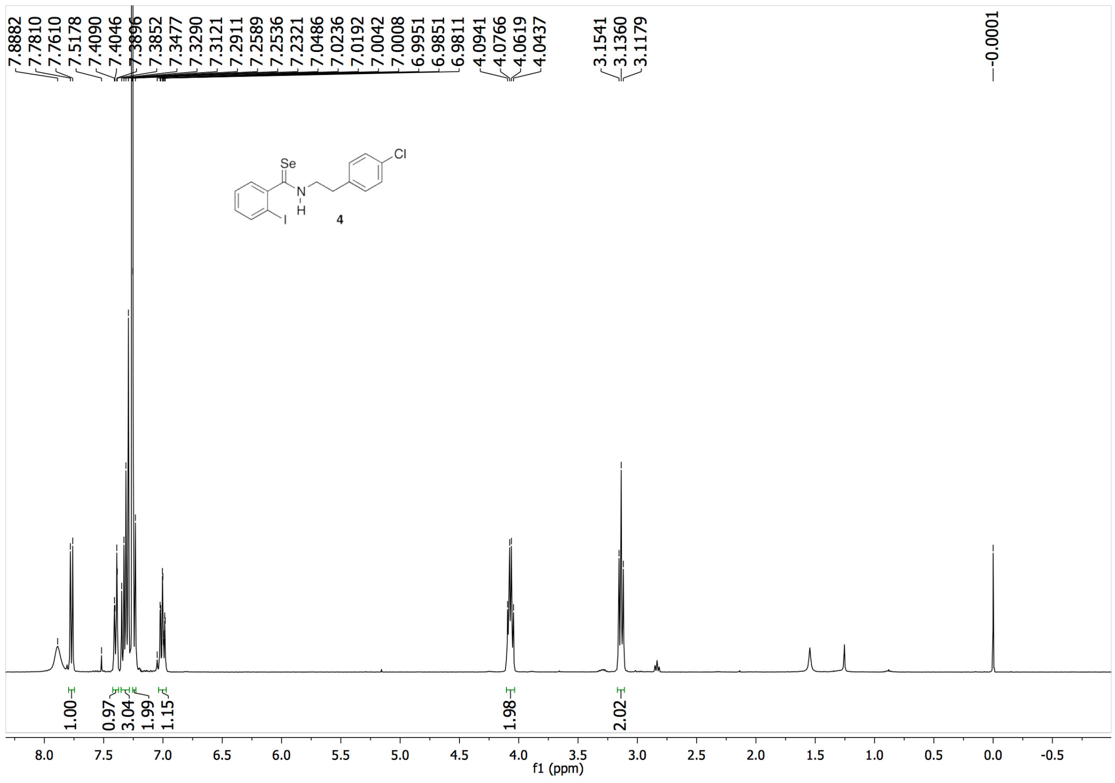

$^{13}$C-NMR ($CDCl_3$ with 0.05% TMS, 100 MHz)

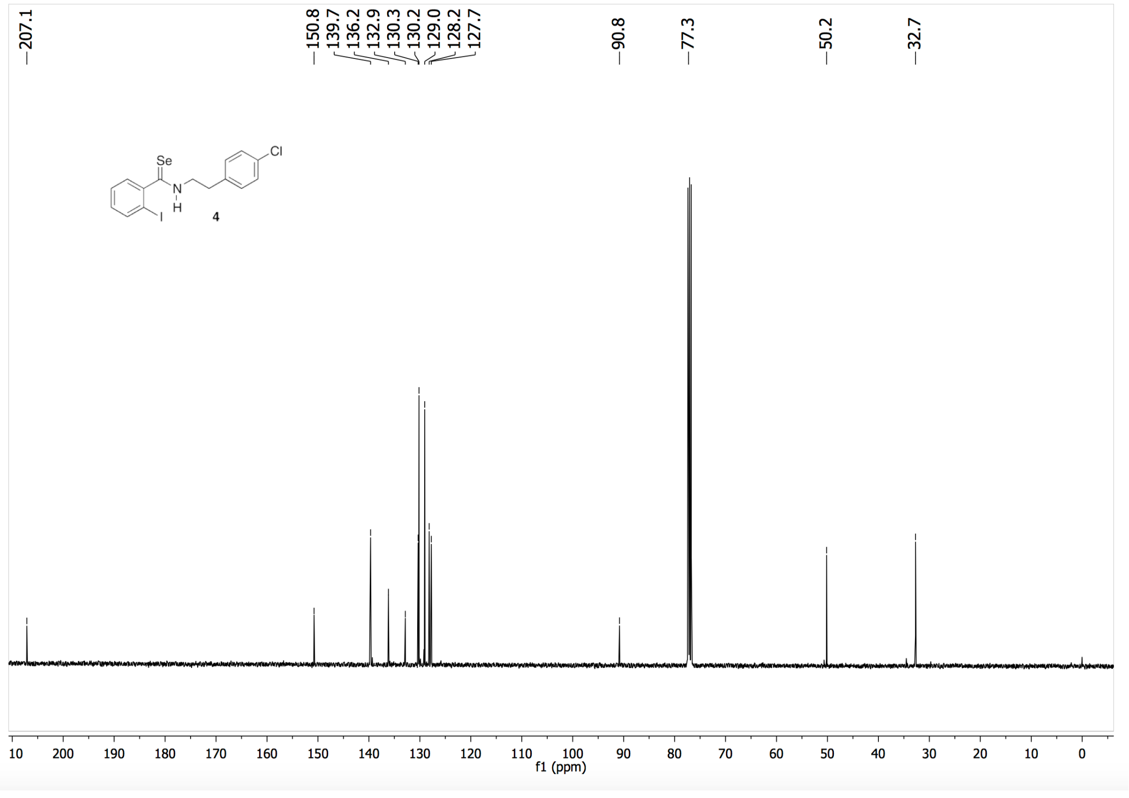

$^{1}$H-NMR ($CDCl_3$ with 0.05% TMS, 400 MHz)

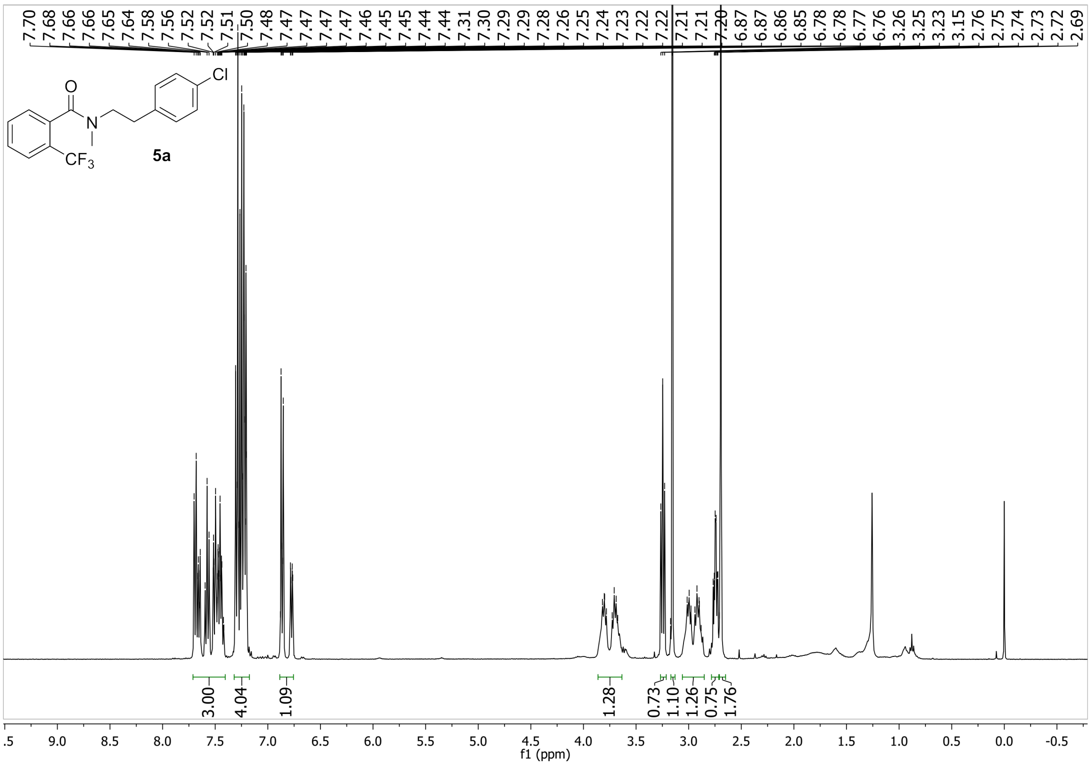


$^{13}$C-NMR ($CDCl_3$ with 0.05% TMS, 100 MHz)

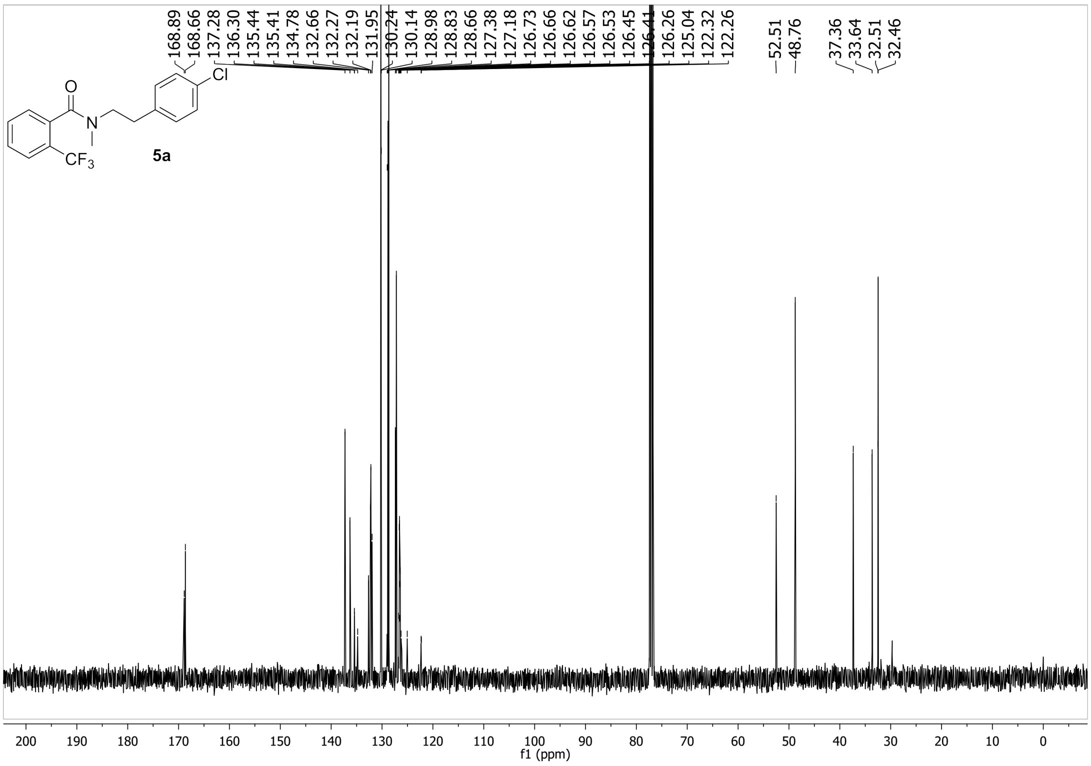

$^{1}$H-NMR ($CDCl_3$ with 0.05% TMS, 400 MHz)

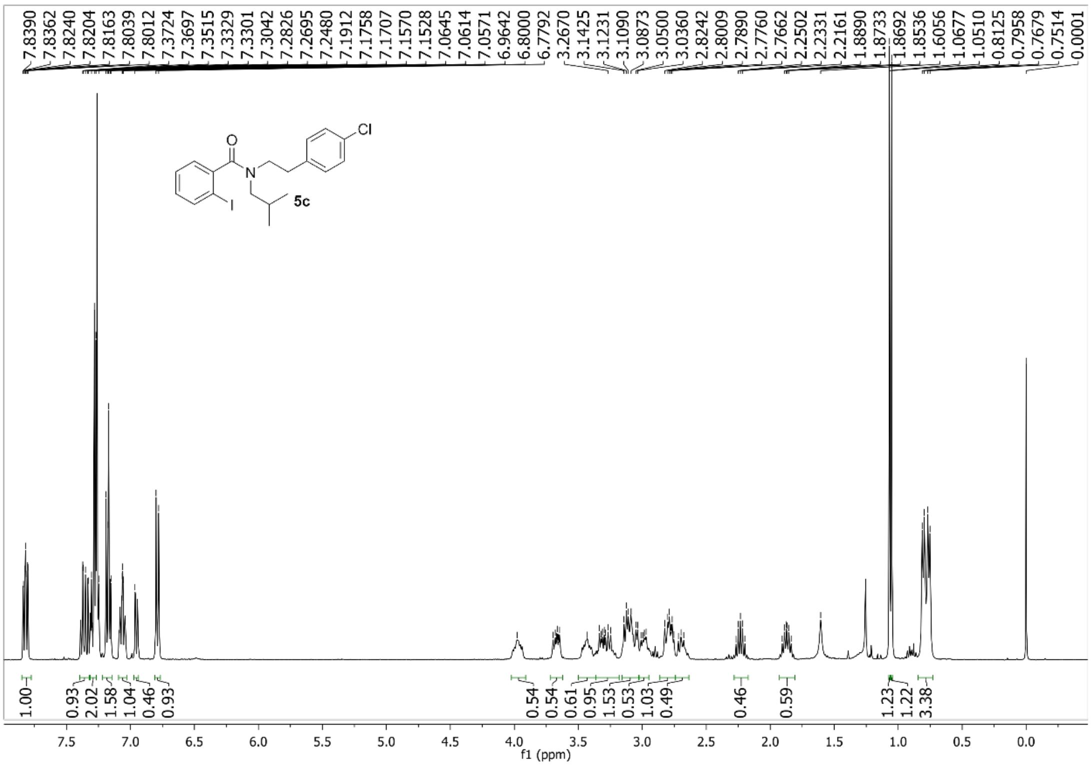


$^{13}$C-NMR ($CDCl_3$ with 0.05% TMS, 100 MHz)

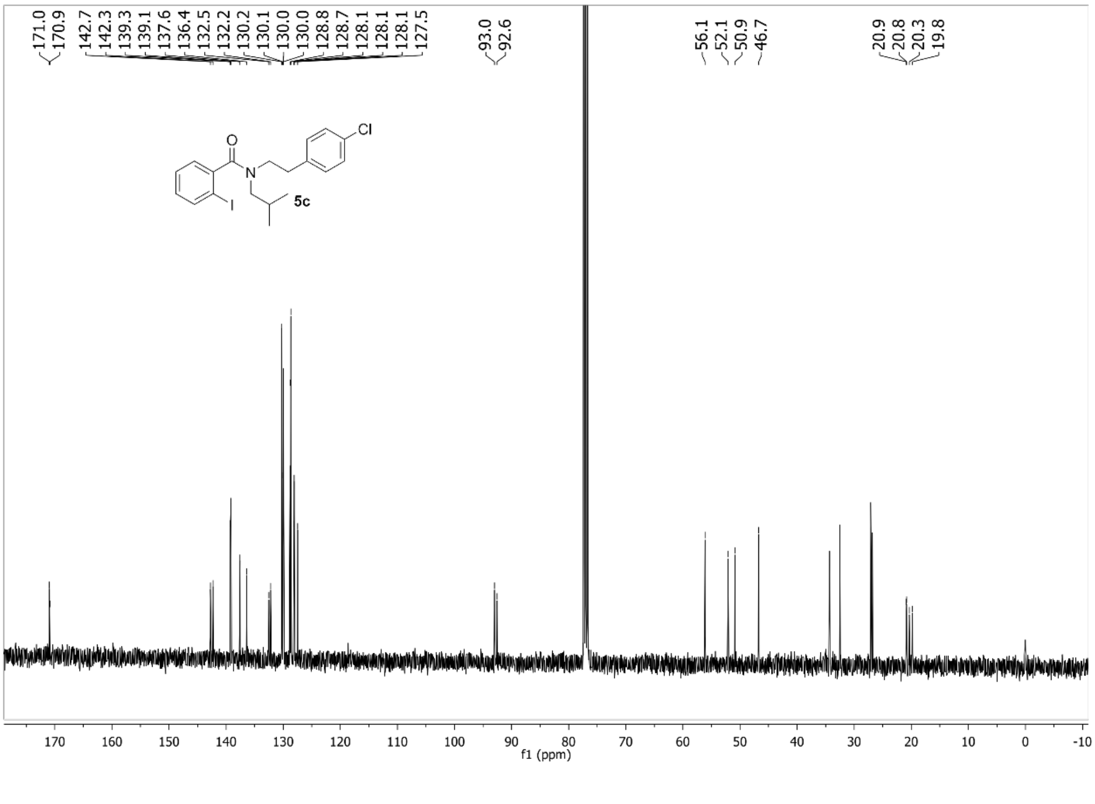

$^1$H-NMR ($CDCl_3$ with 0.05% TMS, 400 MHz)

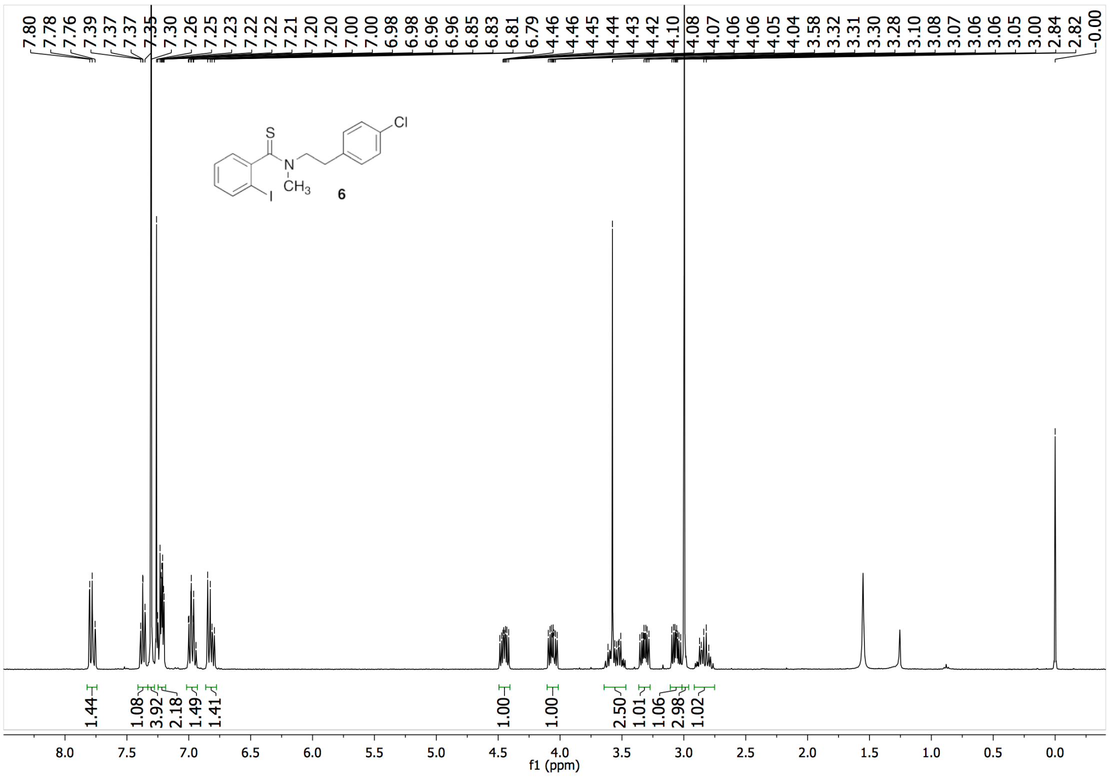

$^{13}$C-NMR ($CDCl_3$ with 0.05% TMS, 100 MHz)

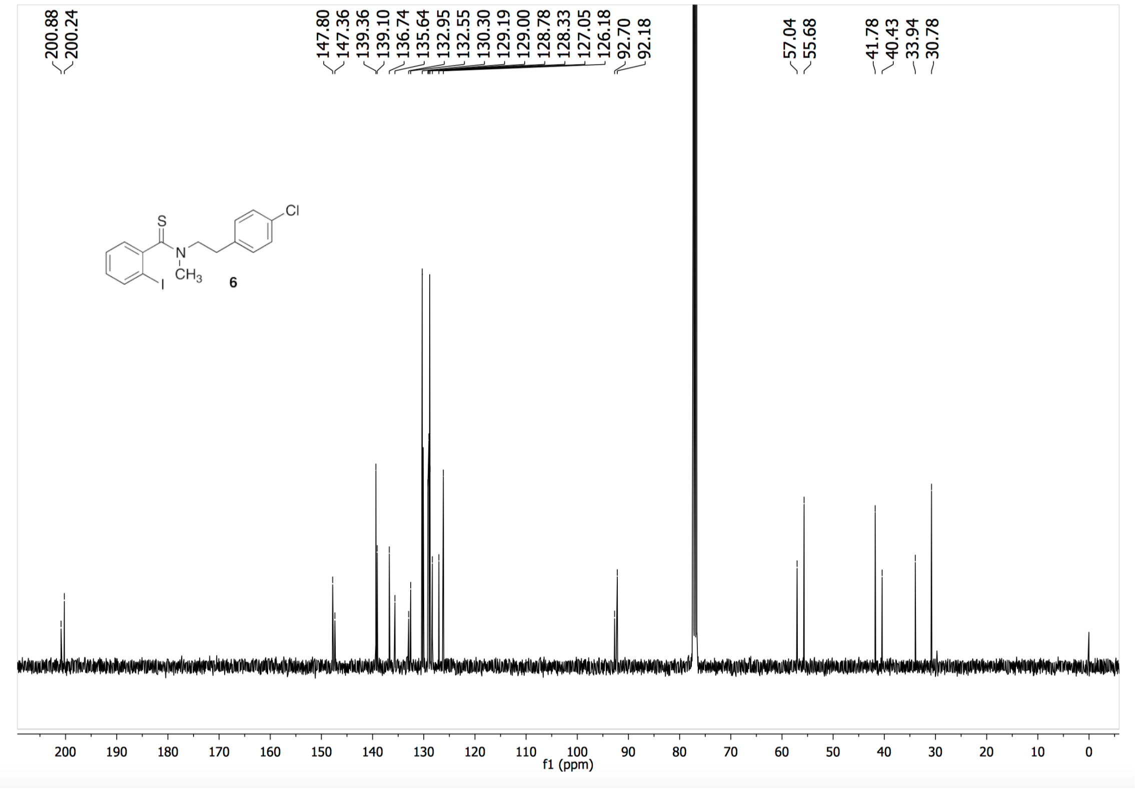

$^1$H-NMR ($(CD_3)_2CO$ with 0.05% TMS, 400 MHz)

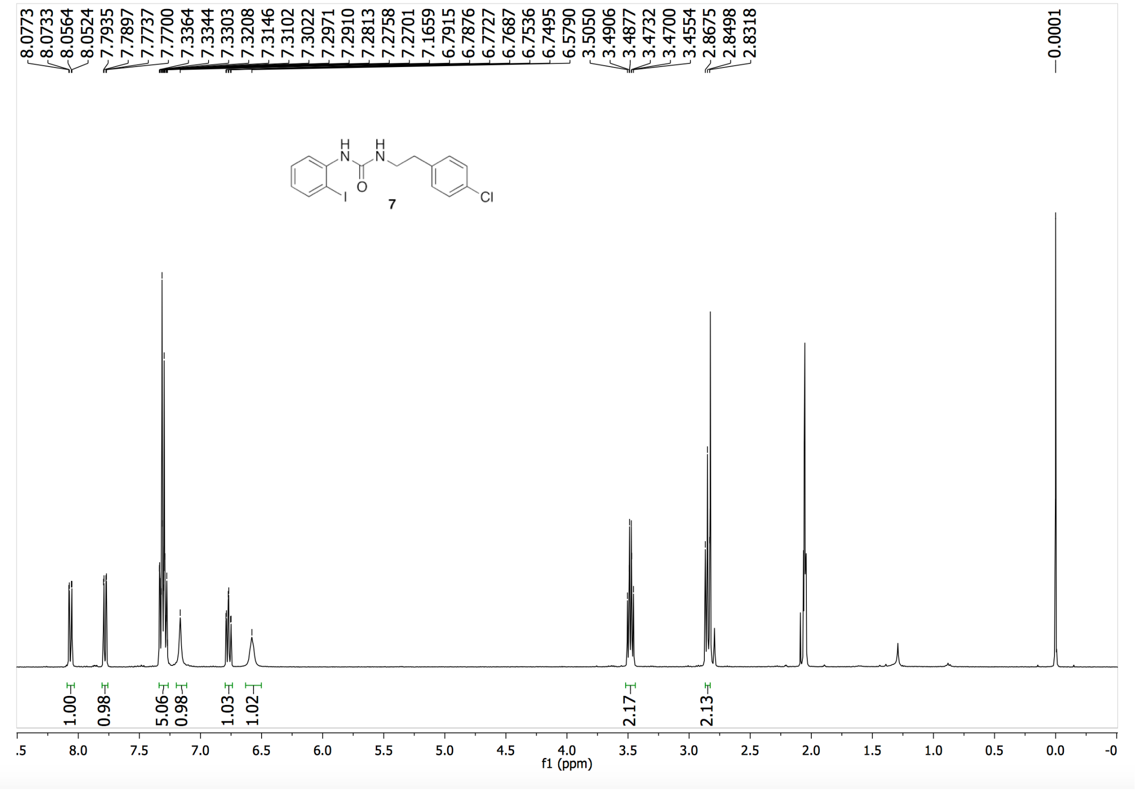


$^{13}$C-NMR ($(CD_3)_2CO$ with 0.05% TMS, 100 MHz)

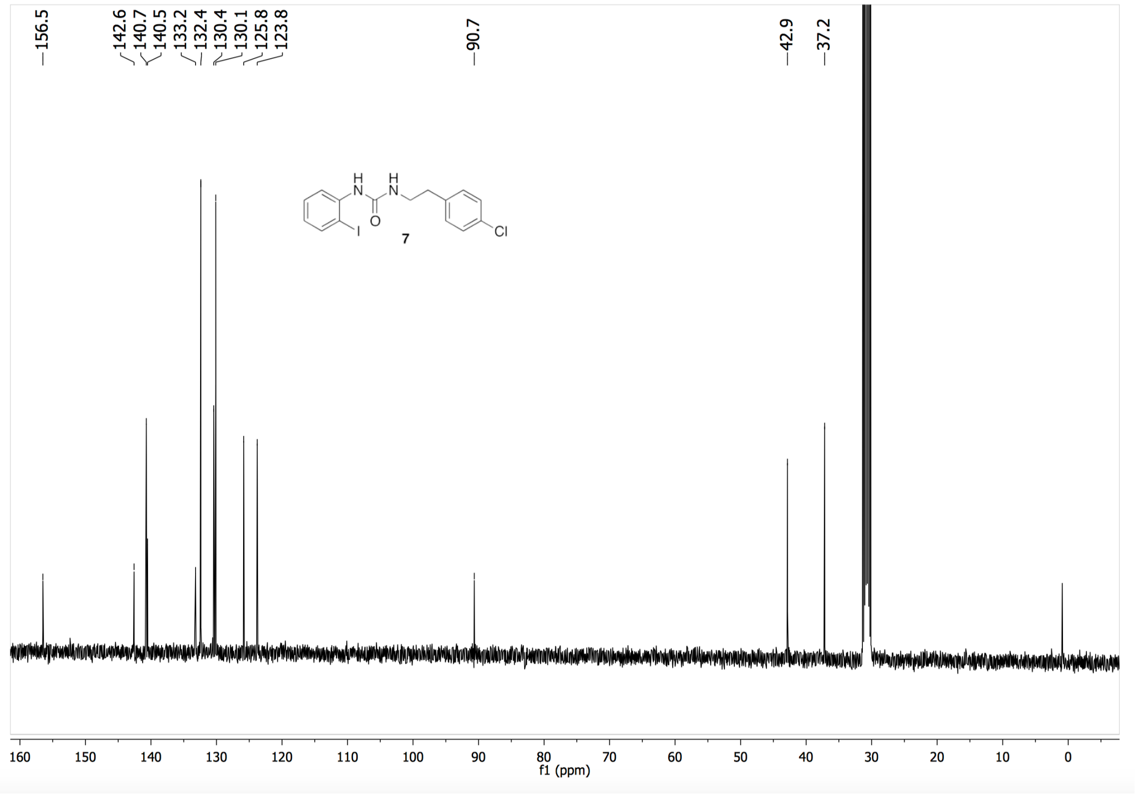

$^{1}$H-NMR ($CDCl_3$ with 0.05% TMS, 400 MHz)

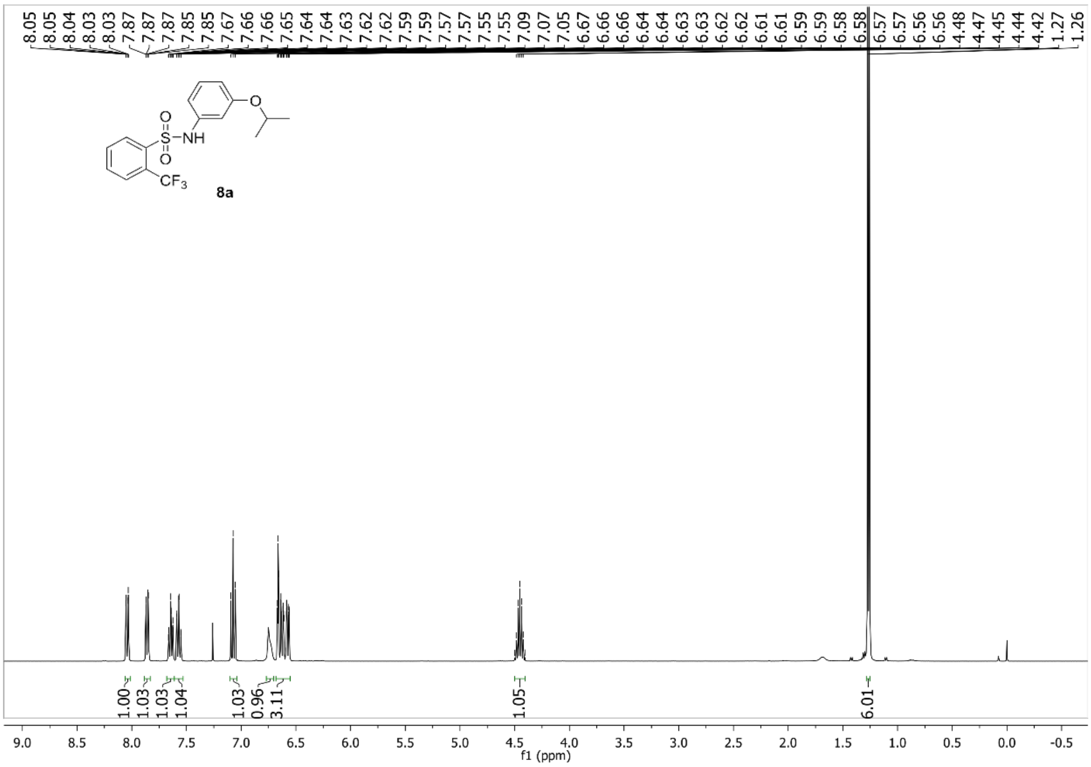

$^{13}$C-NMR ($CDCl_3$ with 0.05% TMS, 100 MHz)

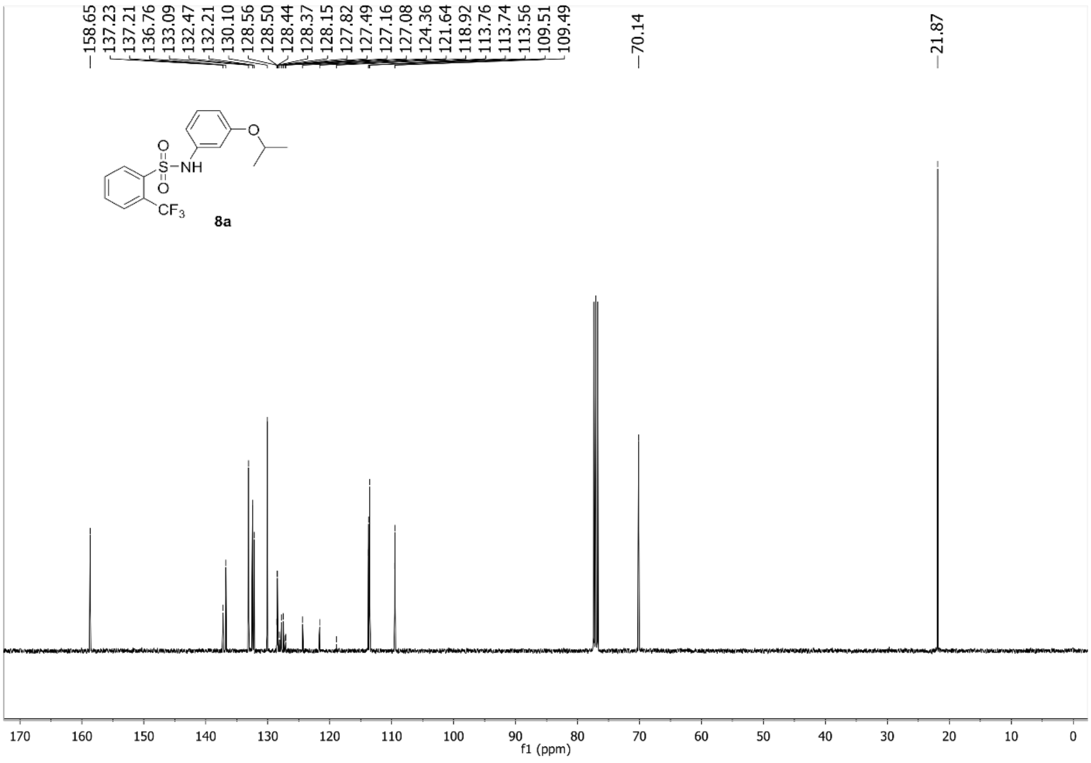

$^{1}$H-NMR ($CDCl_3$ with 0.05% TMS, 400 MHz)

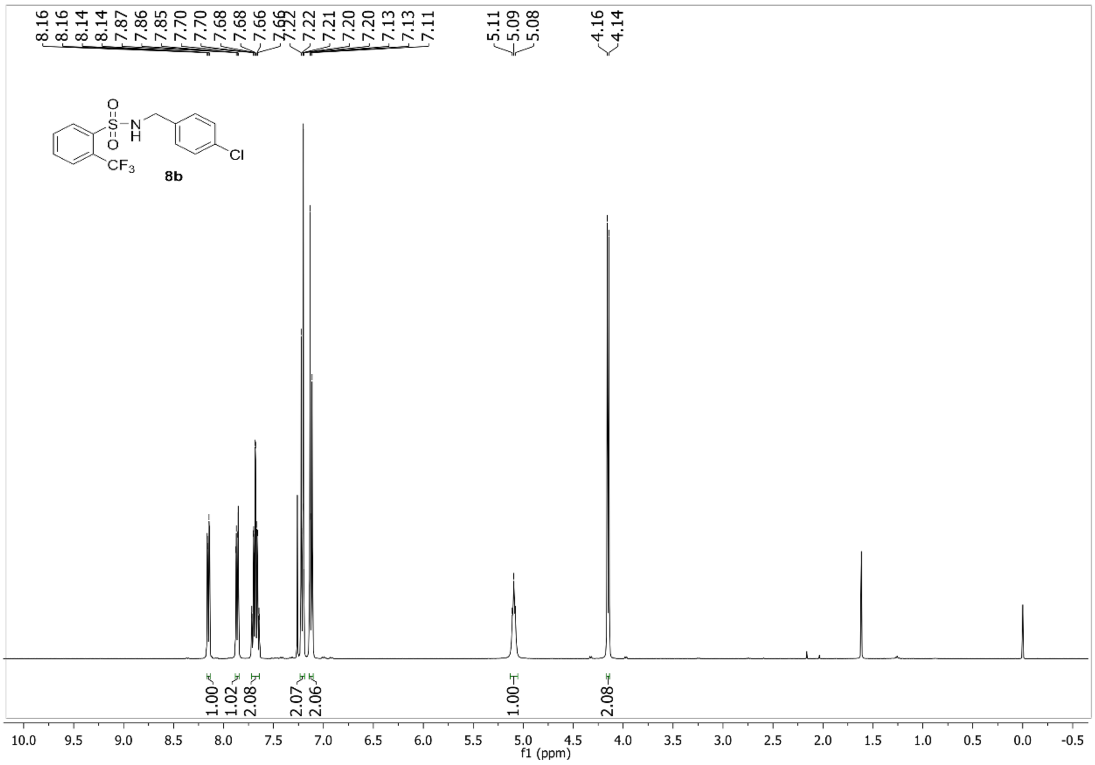


$^{13}$C-NMR ($CDCl_3$ with 0.05% TMS, 100 MHz)

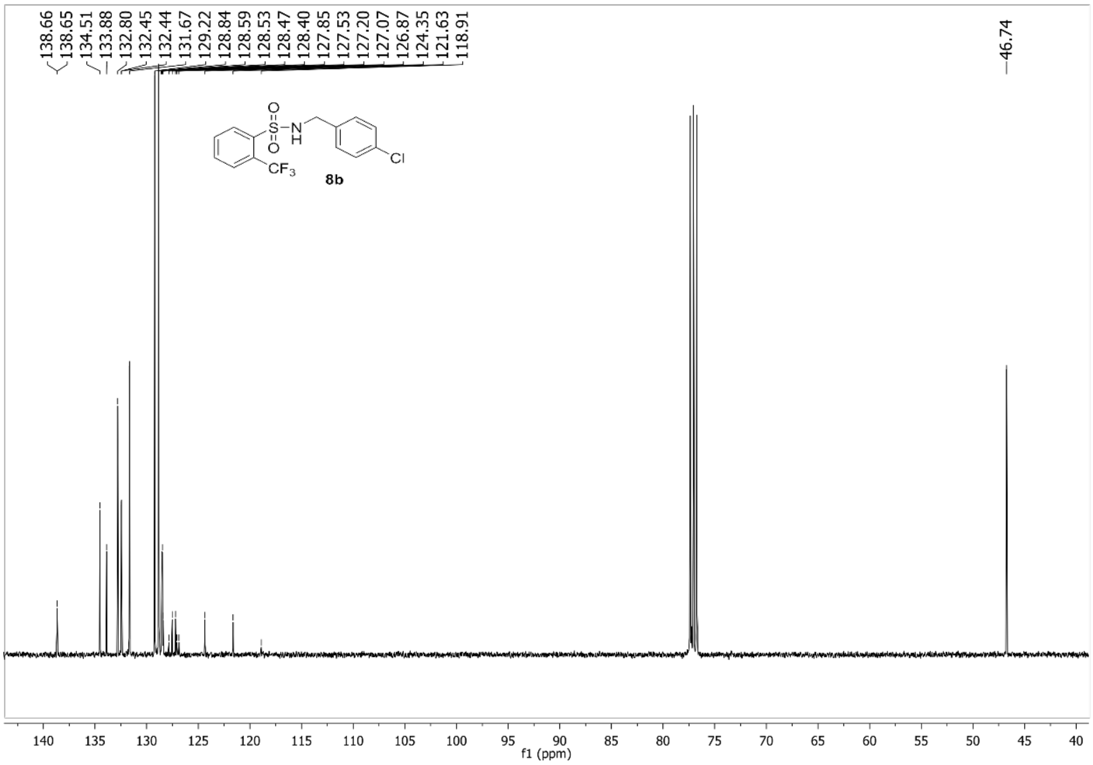

$^{1}$H-NMR ($CDCl_3$ with 0.05% TMS, 400 MHz)

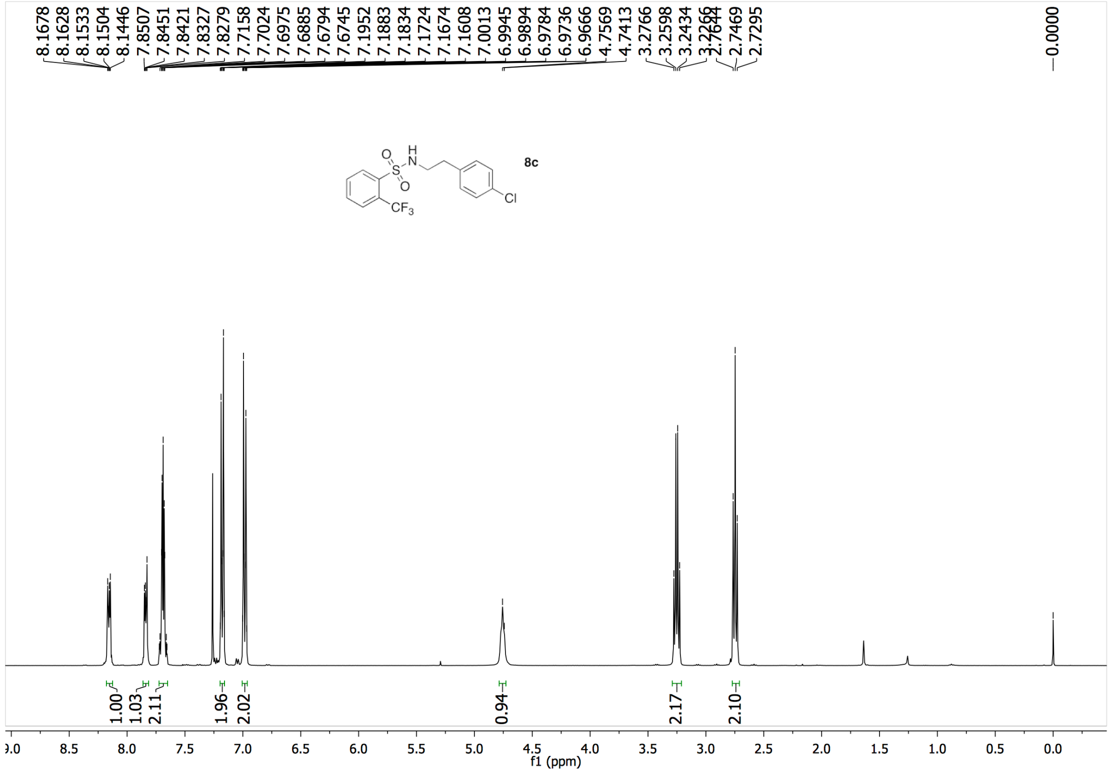


$^{13}$C-NMR ($CDCl_3$ with 0.05% TMS, 100 MHz)

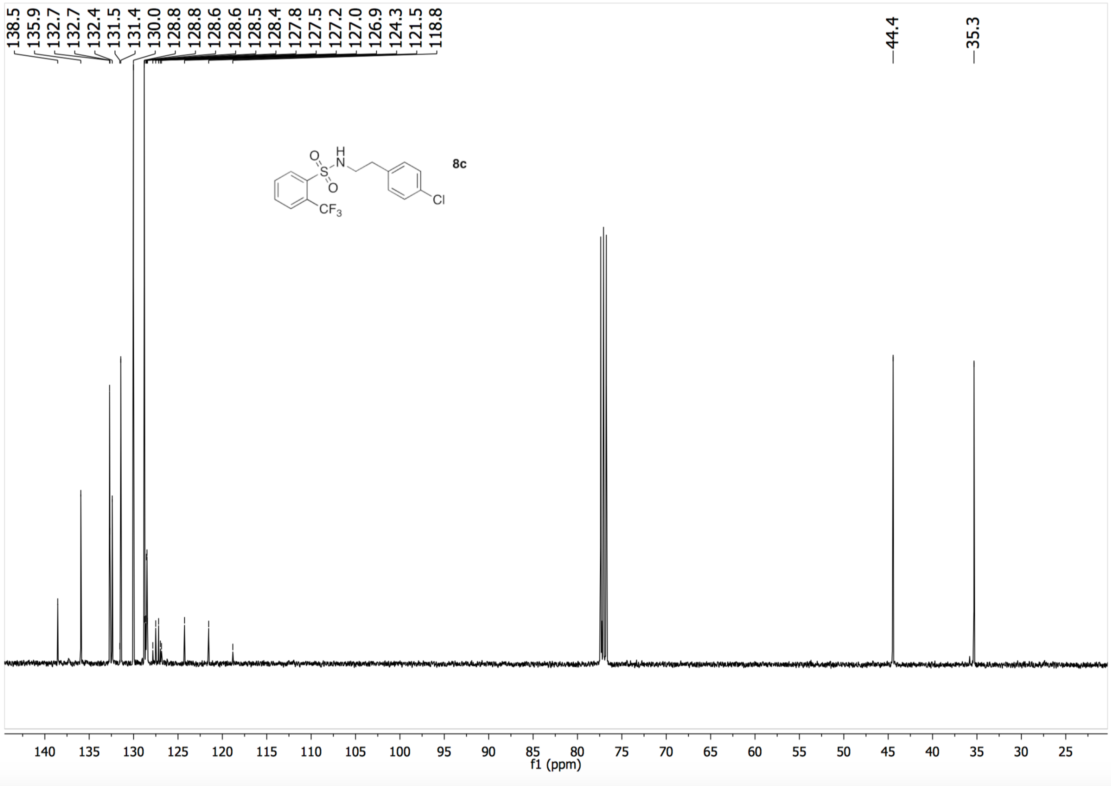

$^{1}H$-NMR ($CDCl_3$ with 0.05% TMS, 400 MHz)

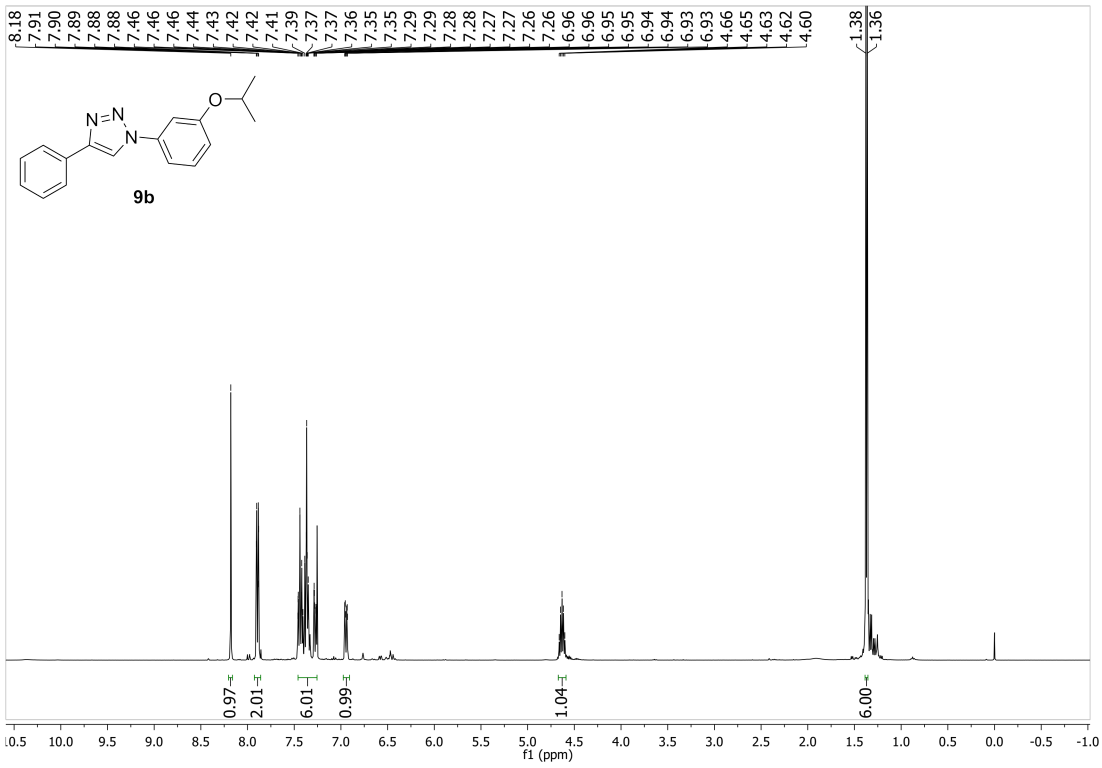

$^{13}C$-NMR ($CDCl_3$ with 0.05% TMS, 100 MHz)

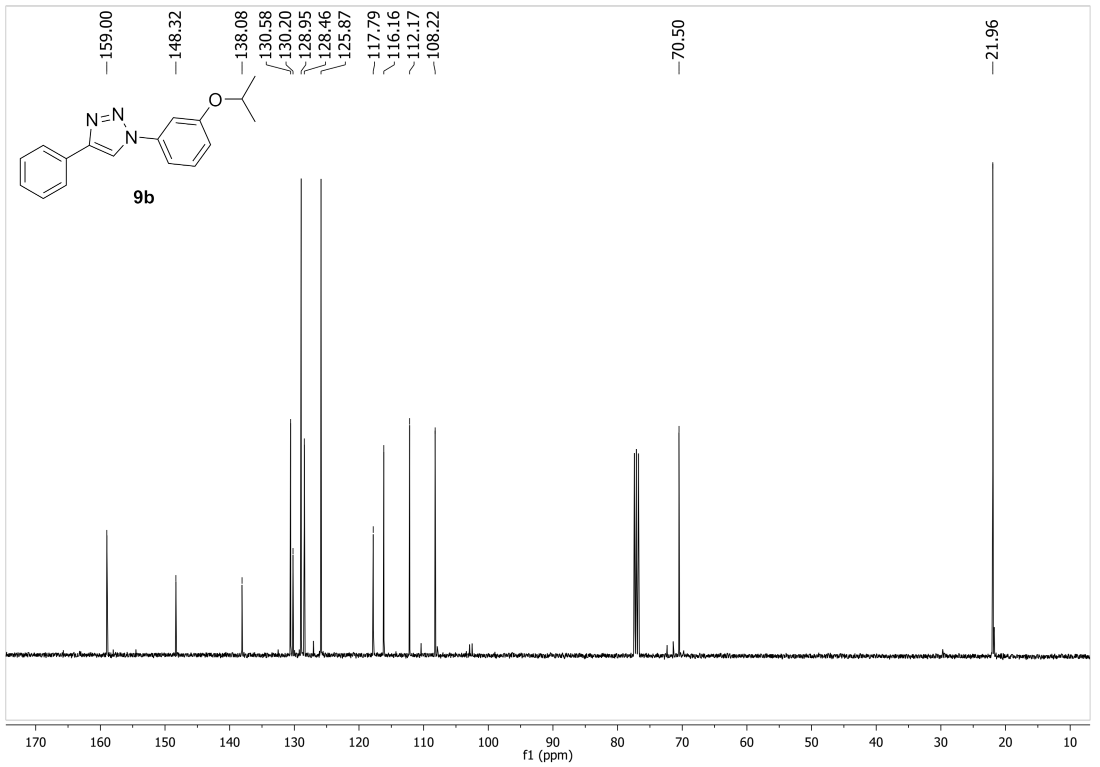

$^{1}$H-NMR ($CDCl_3$ with 0.05% TMS, 400 MHz)

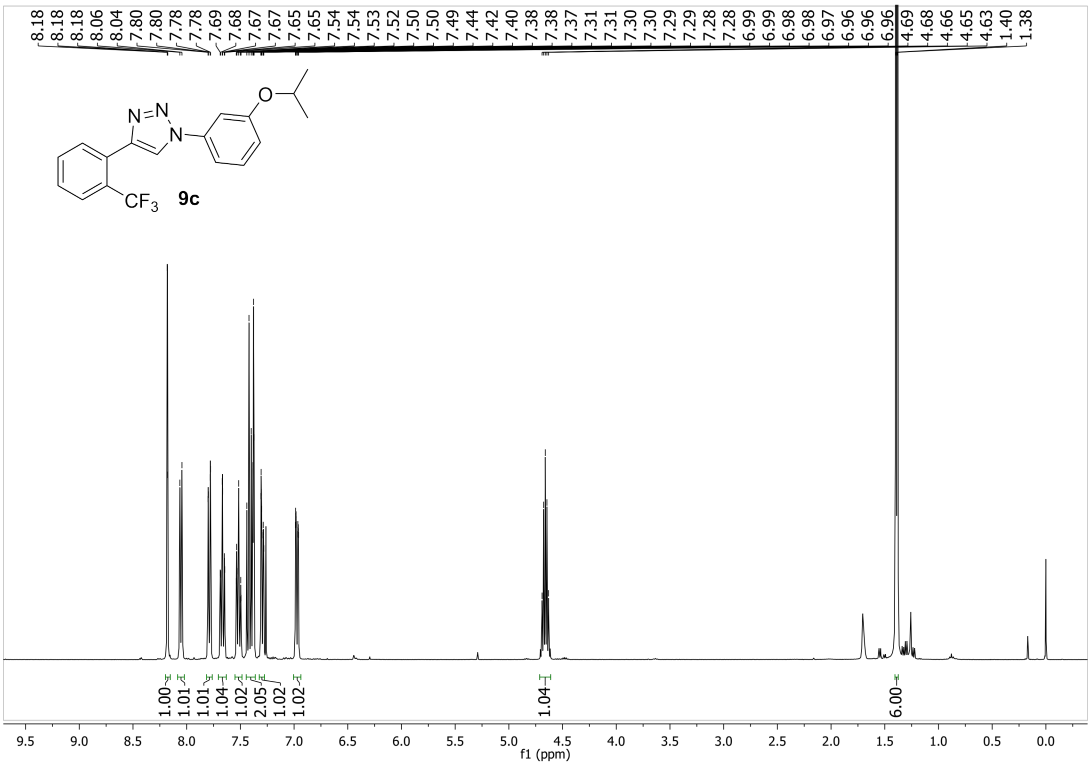


$^{13}$C-NMR ($CDCl_3$ with 0.05% TMS, 100 MHz)

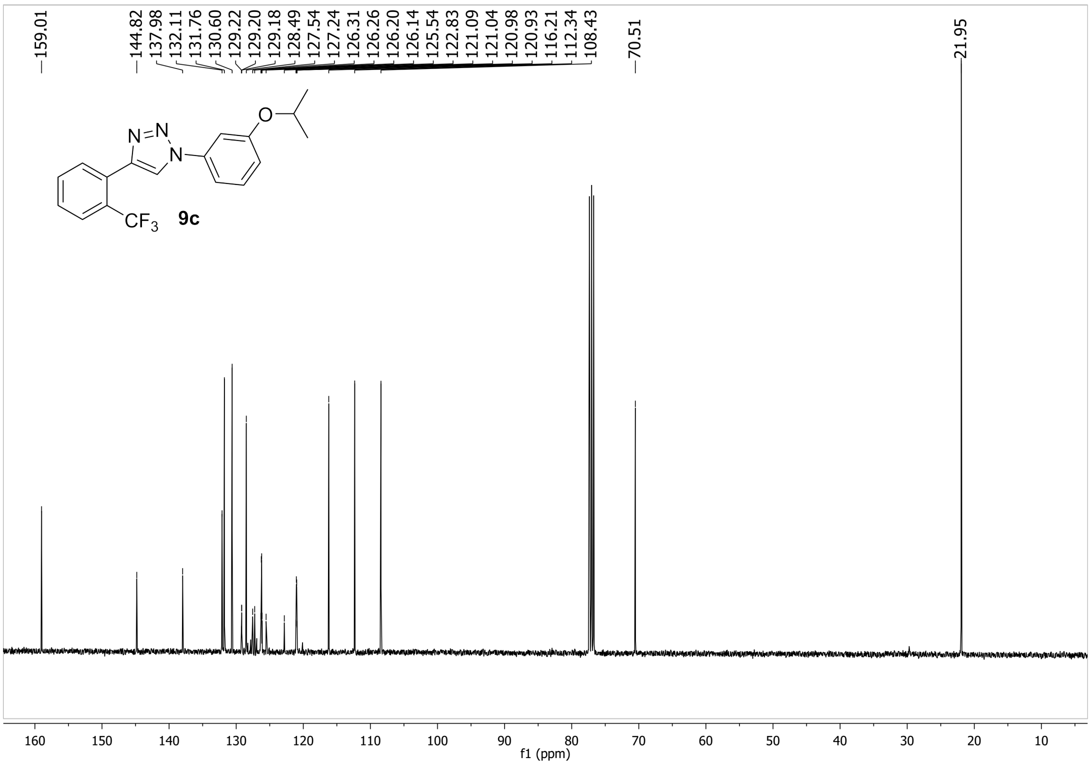

**2. Synthesis and characterization of compounds: 9a, 10b, 11a, 11b** and **11c**.

**(1-(4-chlorobenzyl)-4-phenyl-*1H*-1,2,3-triazole) (9a).** To a solution of **11b** (200 mg, 1.08 mmol) in a *t*-BuOH/$H_2O$ 1:1 mixture (4 mL), phenylacetylene (120 mg, 1.08 mmol), ascorbic acid (40 mg, 0.2 mmol) and $CuSO_4{\cdot}5H_2O$ (5 mg, 0.02 mmol) were added. The mixture was stirred for 24 h and then poured into water (10 mL) and extracted with $CH_2Cl_2$ (3 x 20 mL) Combined organic layers were dried, filtered and removed under reduced pressure. The residue was purified by flash column chromatography on silica gel (EtOAc) to yield **9a** (270 mg, 93%) as a white solid. $^1$H NMR ($CDCl_3$): δ 5.53 (s, 2H), 7.23 (m, 2H), 7.35 (m, 5H), 7.67 (s, 1H), 7.79 (m, 2H). $^{13}$C NMR ($CDCl_3$): δ 53.5, 119.5, 125.7, 128.3, 128.9, 129.4, 130.4, 133.2, 134.9, 148.4. Spectroscopic data is in accordance with that previously reported [58].

**Trimethyl((2-(trifluoromethyl)phenyl)ethynyl)silane) (10b)**. Under argon atmosphere, $Et_3N$ was degassed by bubbling argon for 10 minutes. Ethynyl trimethylsilane (508 mg, 2.2 mmol) and CuI (4 mg, 0.2 mmol) were added and the mixture was stirred for 5 min. 1-yodo-2-(trifluoromethyl)benzene (500 mg, 1.8 mmol) and Pd[P(Ph)$_3$] (42 mg, 0.04 mmol) were added and stirred for 18 h. The mixture was poured into water, acidified to pH 2 with HCl 5% and extracted with EtOAc (3 x 20 mL). The combined organic layers were dried with $Na_2SO_4$, filtered and the solvent was removed under reduced pressure. The residue was purified by flash column chromatography on silica gel (Hx/EtOAc (100:1)) yielding **10b** (270 mg, 61%) as a colourless oil. Rf (Hx) = 0.7. $^1$H NMR ($CDCl_3$): δ 0.26 (s, 9H), 7.37 (dddd, *J* = 8.4, 7.7, 1.5, 0.8 Hz, 1H), 7.45 (tdd, *J* = 7.6, 1.5, 0.7 Hz, 1H), 7.60 (quint, $J_{CF}$ = 7.7, 1.4). $^{13}$C NMR ($CDCl_3$): δ 101.0, 101.4, 121.9 (q, J = 2.2 Hz), 123.9 (d, J $J_{CF}$ = 273.4 Hz) ($CF_3$), 126.2 (q, $J_{CF}$ = 5.1 Hz), 128.6, 131.7, 132.6 (q, J = 30.5 Hz), 134.6 [59].

**1-(azidomethyl)-4-chlorobenzene. (11a)** To a solution of *p*-chlorobenzylamine (500 mg, 3.5 mmol) in 10 mL of MeOH, $K_2CO_3$ (880 mg, 1.28 mmol), $CuSO_4{\cdot}5H_2O$ (10 mg, 0.05 mmol) and 1*H*-imidazole-1-sulfonyl azide hydrochloride (878 mg, 4.2 mmol) were added. The mixture was stirred overnight and then poured into water, acidified to pH 2 with HCl 5% and extracted with $CH_2Cl_2$ (3 x 20 mL). The combined organic layers were dried with $Na_2SO_4$, filtered and removed under reduced pressure. The crude was purified by flash column chromatography on silica gel (Hx/EtOAc (8:1)), yielding **11a** (490 mg, 83%) as a colourless oil. $^1$H NMR ($CDCl_3$): δ 4.29 (s, 2H), 7.23 (m, 2H), 7.34 (m, 2H). $^{13}$C NMR ($CDCl_3$): δ 54.1, 129.1, 129.5, 133.9, 134.2 [60].

**1-(2-azidoethyl)-4-chlorobenzene (11b)** To a solution of 2-(4-chlorophenyl)ethan-1-amine) (100 mg, 0.64 mmol) in MeOH (2 mL) 1*H*-imidazole-1-sulfonyl azide hydrochloride (160 mg, 0.74 mmol), $CuSO_4{\cdot}5H_2O$ (2.2 mg, 0.01 mmol) and anhydrous $K_2CO_3$ (176 mg, 1.28 mmol) were added. The mixture was stirred at room temperature overnight. The solvent was removed at reduced pressure and the residue was poured into water, acidified to pH 2 with HCl 5% and extracted with $CH_2Cl_2$ (3 x 20 mL). The combined organic layers were dried with $Na_2SO_4$, filtered and the solvent was removed under reduced pressure. The crude was purified by flash column chromatography on silica gel (Hx/EtOAc (8:1)), yielding **11b** (102 mg, 88%) as a colourless oil. $^1$H NMR ($CDCl_3$): δ 2.84 (t, *J* = 7.1 Hz, 2H), 3.47 (t, *J* =7.1 Hz, 2H), 7.14 (m, 2H), 7.28 (m, 2H). $^{13}$C NMR ($CDCl_3$): δ 34.7, 52.3, 128.8, 130.1, 132.7, 136.6 [40-62].

**1-azide-3-isopropoxybenzene. (11c)** To a solution of 3-isopropoxyaniline (200 mg, 1.32 mmol) in HCl 6 N (5 mL), $NaNO_2$ (91 mg, 1.32 mmol) was added in ice bath. The mixture was stirred at room temperature for 10 min and $NaN_3$ (103 mg, 1.6 mmol) was added. After 2 h, the mixture was poured into water, neutralized with $NaHCO_3$ 10% and extracted with EtOAc (3 x 15 mL). The combined organic layers were dried with $Na_2SO_4$, filtered and removed under reduced pressure. The reaction crude was used in the next step without further purification [63].

## 3. Docking optimization

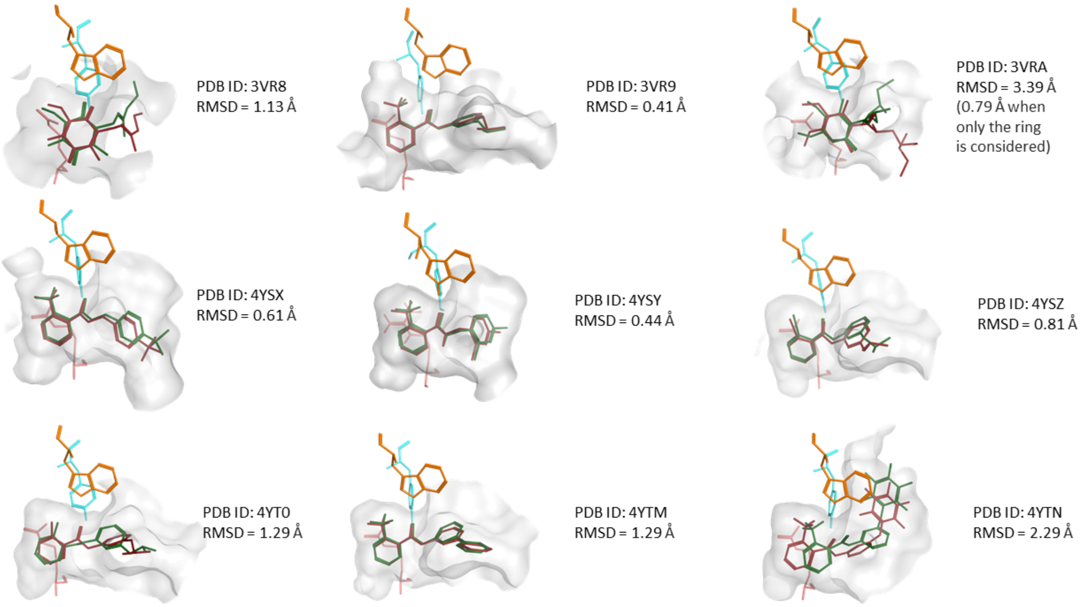


**Supplementary Figure S1**. Docking optimization. *A.suum* Complex II structures deposited in PDB were retrieved to check the ability of the docking protocol to replicate experimental binding modes of complex II Q-site ligands. For each ligand, the crystallographic pose is shown in green superimposed with the docking pose with best S score, depicted in red. Receptor molecular surface and relevant interacting residues (TRP251B in orange, TYR96D in cyan and ARG76C in pink) are shown. In all cases the docking pose is in agreement with the crystallographic one and except for 4YTN, all satisfy the RMSD criteria. Cross docking experiments yielded similar results.

## 4. Docking poses obtained for Wact-11 bioisostere derivatives:

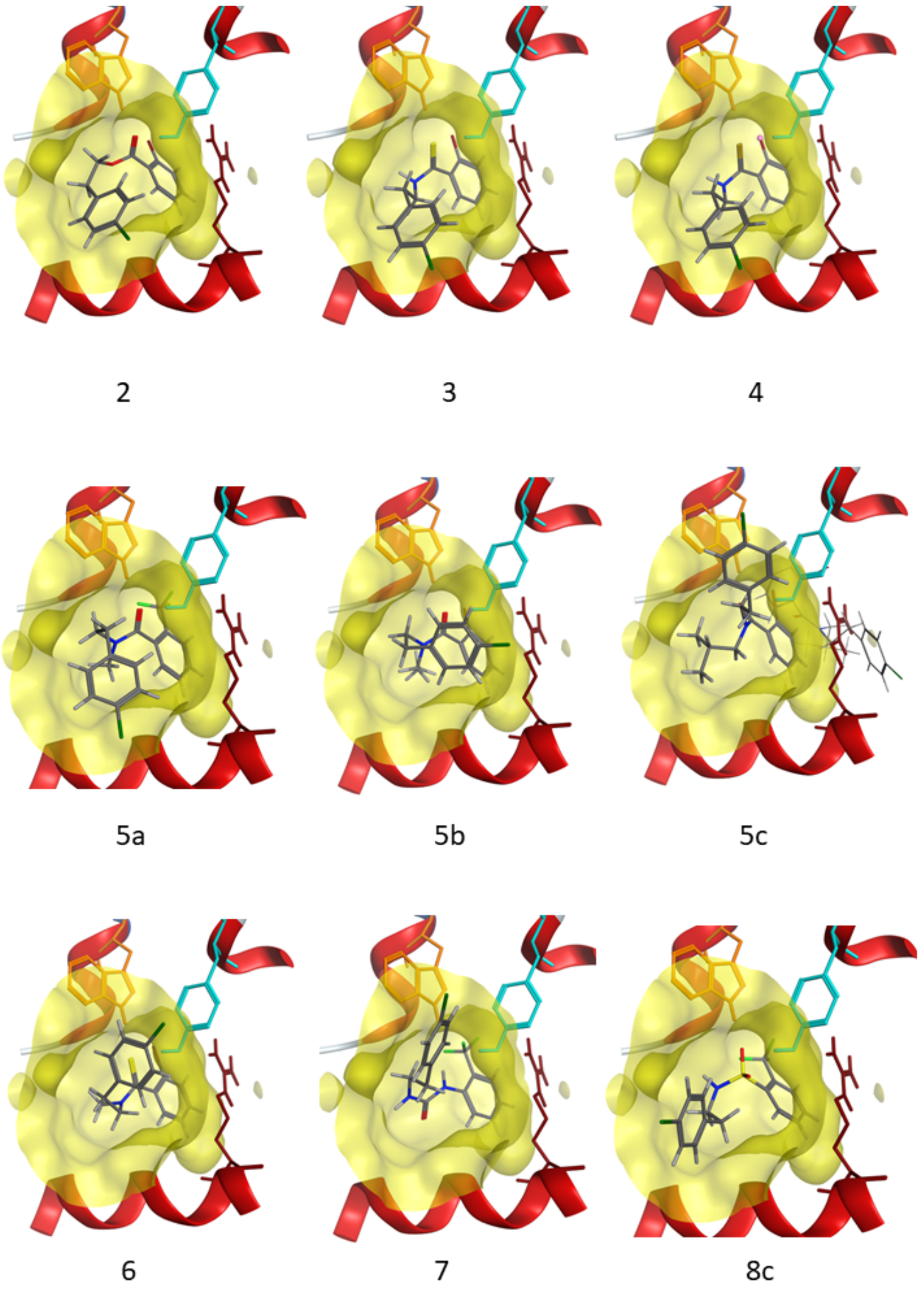


**Supplementary Figure S2.** Docking poses obtained for Wact-11 bioisostere derivatives **2-8c.**

## 5. Interaction diagrams for compounds 3 and 4.

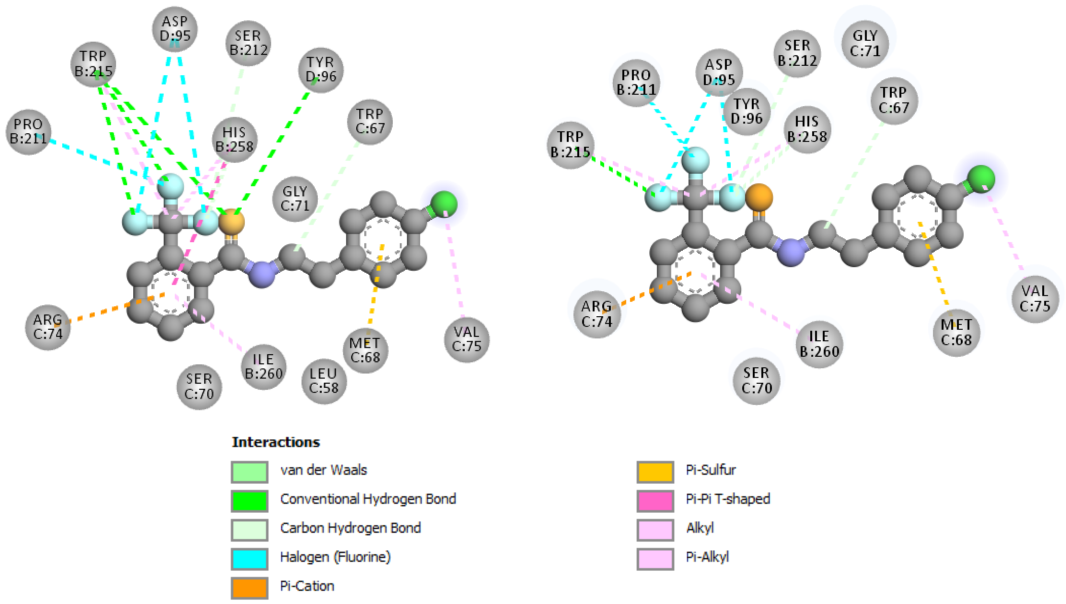


**Supplementary Figure S3.** Interaction diagram for **3** (left) and **4** (right) docked in *C. elegans* complex II, generated in Discovery Studio Visualizer 16, Biovia Corp.

## 6. Simulated mitochondrial membrane with Fluopyram.

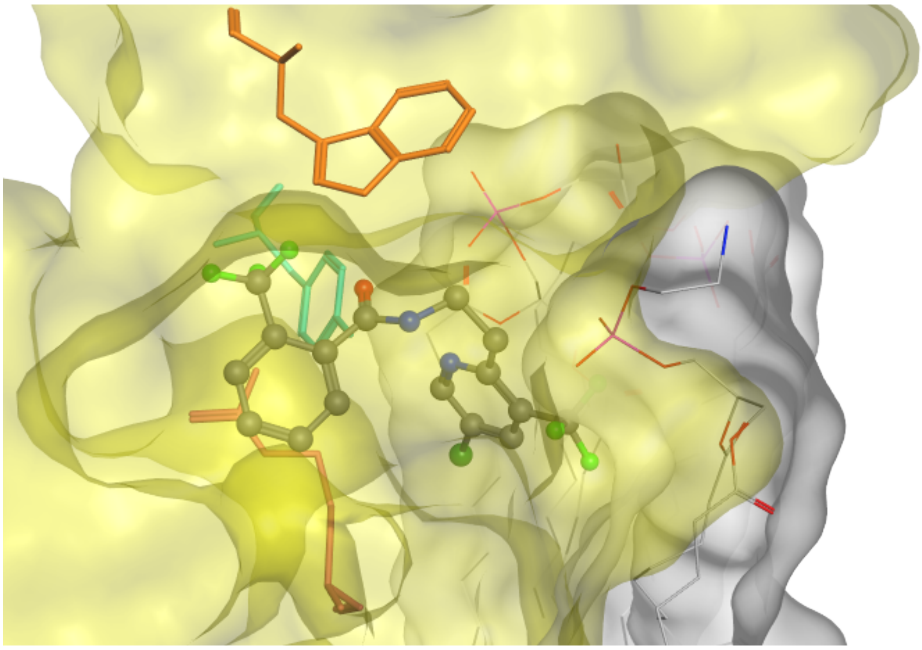

**Supplementary Figure S4.** Fluopyram docked in *C. elegans* complex II. Main interacting residues are shown (Trp215 of subunit B in orange, Tyr96 of subunit D in cyan and Arg74 of subunit C in pink). The pose is representative of that obtained for all benzamides.

**7. *C. elegans* L1 motility assay results for compounds 8a, 8c, 9a-c.**

| Entry | Compound | Structure | % *C. elegans* motility reduction (10 μM)[a] |
|---|---|---|---|
| 1 | **8a** | 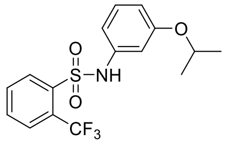 | 0 |
| 2 | **8b** | 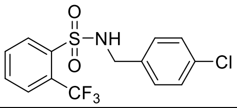 | 2 |
| 3 | **9a** | 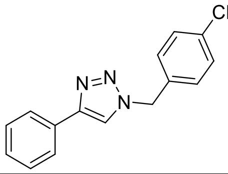 | 0 |
| 4 | **9b** | 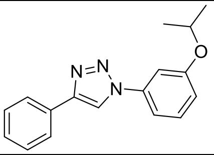 | 0 |
| 5 | **9c** | 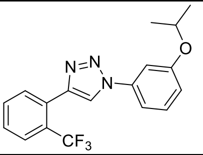 | 4 |

a. Calculated compared to vehicle motility (DMSO 1%) taken as 0% motility reduction. Compounds were evaluated at 10μM after 20 h.

**Supplementary Table S1**. Library screening in *C. elegans* L1 of non-Wact11 analogous compounds.
